# Biofilm is a Major Virulence Determinant in Bacterial Colonization of Chronic Skin Ulcers Independently from the Multidrug Resistant Phenotype

**DOI:** 10.3390/ijms18051077

**Published:** 2017-05-17

**Authors:** Enea Gino Di Domenico, Ilaria Farulla, Grazia Prignano, Maria Teresa Gallo, Matteo Vespaziani, Ilaria Cavallo, Isabella Sperduti, Martina Pontone, Valentina Bordignon, Laura Cilli, Alessandra De Santis, Fabiola Di Salvo, Fulvia Pimpinelli, Ilaria Lesnoni La Parola, Luigi Toma, Fabrizio Ensoli

**Affiliations:** 1 Clinical Pathology and Microbiology, San Gallicano Institute, Istituto di Ricovero e Cura a Carattere Scientifico (IRCCS), 00144 Rome, Italy; ilariafarulla@libero.it (I.F.); grazia.prignano@ifo.gov.it (G.P.); mariateresa.gallo@ifo.gov.it (M.T.G.); mvespaziani@yahoo.it (M.V.); ilaria.cavallo90@gmail.com (I.C.); martinapontone@hotmail.it (M.P.); valentina.bordignon@ifo.gov.it (V.B.); laura81cl@libero.it (L.C.); alessandra21@libero.it (A.D.S.); fabiola-91@hotmail.it (F.D.S.); fulvia.pimpinelli@ifo.gov.it (F.P.); fabrizio.ensoli@ifo.gov.it (F.E.); 2 Biostatistics, San Gallicano Institute, Istituto di Ricovero e Cura a Carattere Scientifico (IRCCS), 00144 Rome, Italy; isabella.sperduti@ifo.gov.it; 3 Department of Dermatology, San Gallicano Institute, Istituto di Ricovero e Cura a Carattere Scientifico (IRCCS), 00144 Rome, Italy; ilaria.lesnoni@ifo.gov.it; 4 Infectious Disease Consultant, Regina Elena National Cancer Institute, Istituto di Ricovero e Cura a Carattere Scientifico (IRCCS), 00144 Rome, Italy; luigi.toma@ifo.gov.it

**Keywords:** skin ulcer, MDRO, biofilm, wound, MRSA, ESBL, *Pseudomonas aeruginosa*, *Klebsiella pneumoniae*, *Escherichia coli*, *Acinetobacter baumannii*

## Abstract

Bacterial biofilm is a major factor in delayed wound healing and high levels of biofilm production have been repeatedly described in multidrug resistant organisms (MDROs). Nevertheless, a quantitative correlation between biofilm production and the profile of antimicrobial drug resistance in delayed wound healing remains to be determined. Microbial identification, antibiotic susceptibility and biofilm production were assessed in 135 clinical isolates from 87 patients. Gram-negative bacteria were the most represented microorganisms (60.8%) with MDROs accounting for 31.8% of the total isolates. Assessment of biofilm production revealed that 80% of the strains were able to form biofilm. A comparable level of biofilm production was found with both MDRO and not-MDRO with no significant differences between groups. All the methicillin-resistant *Staphylococcus aureus* (MRSA) and 80% of *Pseudomonas aeruginosa* MDR strains were found as moderate/high biofilm producers. Conversely, less than 17% of *Klebsiella pneumoniae* extended-spectrum beta-lactamase (ESBL), *Escherichia coli*-ESBL and *Acinetobacter baumannii* were moderate/high biofilm producers. Notably, those strains classified as non-biofilm producers, were always associated with biofilm producer bacteria in polymicrobial colonization. This study shows that biofilm producers were present in all chronic skin ulcers, suggesting that biofilm represents a key virulence determinant in promoting bacterial persistence and chronicity of ulcerative lesions independently from the MDRO phenotype.

## 1. Introduction

Wounds are particularly predisposed to microbial colonization and considered at very high risk for multidrug resistant organism (MDRO) infections [1,2,3,4,5]. Clinical/surgical management of chronic wounds accounts for 2–4% of the total health budget in western countries and this estimate is expected to increase due to population aging and to the growing number of people with predisposing factors such as diabetes and obesity [6,7]. Generally, the most common MDROs isolated from patients with chronic ulcerations include methicillin-resistant *S. aureus* (MRSA), bacteria producing extended-spectrum beta-lactamase (ESBL) and carbapenemases (*K. pneumoniae* and *E. coli*), as well as multidrug resistant *P. aeruginosa* (MDRPA) and *A. baumannii* [8]. Nevertheless, the prevalence of MDROs can significantly differ either temporally or geographically, also depending on the specific healthcare setting [4,8,9,10,11]. Consequently, the approaches to prevent and control MDROs should be tailored to the specific needs of each population and institution. Patients with chronic ulcers usually harbor a variety of colonizing bacterial species [12]. An accurate microbiological diagnosis is essential to prevent serious infections and to avoid inappropriate treatment that causes deterioration of the wound, thus delaying healing. Considering that initial treatment is empirical in about two-thirds of the cases, selecting an appropriate empiric antibiotic regimen is critical and requires knowing the usual etiologic organisms and the local prevalence of pathogens, especially antibiotic-resistant strains [13,14].

Complications related to non-healing ulcers are not limited to the presence of MDRO. In fact, increasing evidence suggests that biofilm may represent a major virulence factor in the pathogenesis of chronic ulceration [15,16,17]. The term “critical colonization” defines a condition in which the host defenses are compromised and bacteria induce the failure of wounds healing in the absence of signs of systemic infection [18,19]. Thus, “critical colonization” is the clinical condition that better identifies the putative role of bacterial biofilm in a chronic wound [20]. Indeed, chronic ulcers offer an ideal environment for biofilm formation due to the reduced host immune response in the wounded area, characterized by the presence of necrotic tissue that, in turn, can promote microbial adhesion [10,21]. This is confirmed by electron microscopy of biopsies revealing that biofilm is present in almost 60% of the samples from chronic wounds in comparison with only 6% of biopsies deriving from patients with acute wounds [22]. On the other hand, bacteria endowed in a mature biofilm matrix, firmly adherent to the surrounding tissues, can effectively escape the host local immune response as well as the conventional antimicrobial treatments. As a result, they are particularly difficult to eradicate since they require a minimum inhibitory concentration (MIC) impossible to reach in vivo, due to the side effects and the toxicity of the drugs at renal and/or hepatic level [23,24,25].

Although several reports described a higher level of biofilm production by MDROs, the correlation between biofilm production and the acquisition of antibiotic resistance is still debated [26,27,28,29]. Numerous studies to date highlight the importance of the bacterial biofilm as a potential virulence factor contributing to microbial invasiveness and persistence. However, biofilm production is not routinely investigated in clinical microbiology testing. In fact, conventional clinical microbiology testing targets only the planktonic microbial forms, which are entirely different from the sessile forms, endowed within the biomass [30,31,32]. Thus, assessment of the biofilm production capabilities represents a necessary prerequisite for the development of novel medical or surgical therapeutic interventions, since, at present, the most effective strategy against biofilm consists of its surgical removal from the wound surface [33].

This study aimed at investigating the spectrum of microbial colonization in patients with chronic ulcers and at evaluating the biofilm production capabilities of drug resistant Gram-positive and Gram-negative bacteria.

## 2. Results

From the 87 patients with a colonized skin ulcer included in the study, 52% percent were men and 48% women, with a mean age of 62 years (standard deviation 16.73, range 23–94). The most relevant pathologies reported were venous leg ulcers in 56.3% of cases, with diabetic ulcerations involved in 18% of cases and pressure ulcer accounting for 25.2%. Other entities included ulcerations secondary to a trauma (7.8%) and neoplasms (5.8%) and arterial leg ulcers described in 4.9% of patients. A total of 135 different bacterial species were isolated from ulcerations. In particular, single bacterial species were isolated from 45 ulcerative lesions, while two different species were observed in 36 lesions. In the remaining six cases, the presence of three different microorganisms was observed.

**Figure 1:**
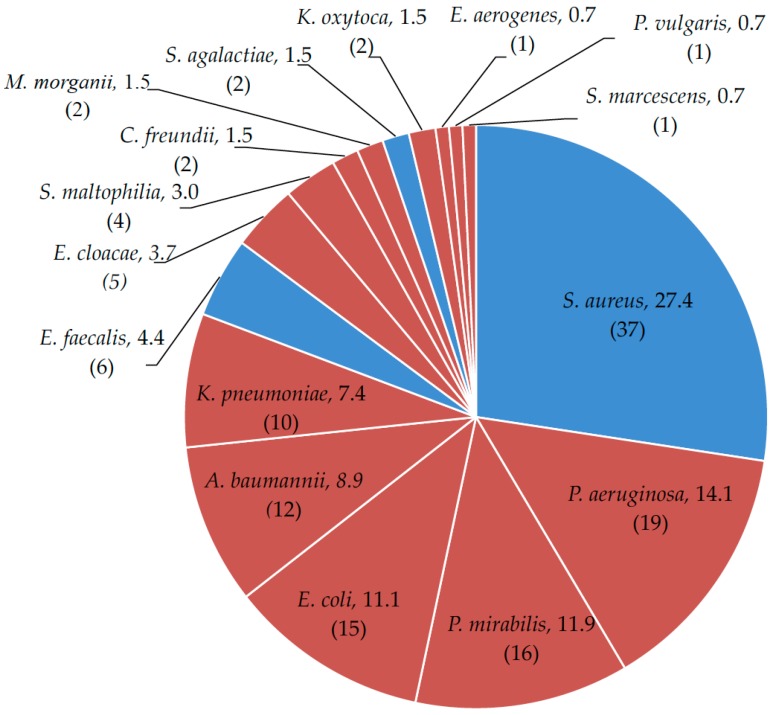
Bacterial isolates from patients with colonized skin ulcers: *Staphylococcus aureus*, *Pseudomonas aeruginosa*, *Proteus mirabilis*, *Escherichia coli*, *Acinetobacter baumannii*, *Klebsiella pneumonia*, *Enterococcus faecalis*, *Enterobacter cloacae*, *Stenotrophomonas maltophilia*, *Citrobacter freundii*, *Morganella morganii*, *Streptococcus agalactiae*, *Klebsiella oxytoca*, *Enterobacter aerogenes*, *Proteus vulgaris* and *Serratia marcescens*. Data in percentage. In brackets, the absolute occurrence of different bacterial species. Blue = Gram-positive; red = Gram-negative.

Globally, sixteen different pathogenic bacterial species were identified. Specifically, Gram-negative bacilli were more frequently (66.7%) isolated from the ulcer swabs than Gram-positive cocci (33.3%). Nevertheless, the Gram-positive *S. aureus* was the most common pathogen isolated, accounting for 27.4% of the total microorganisms (Figure 1). Among the Gram-negative bacteria, the most frequent species was *P. aeruginosa* (14.1%).

In descending order, other species isolated were *Proteus mirabilis* (11.9%), *E. coli* (11.1 %), *A. baumannii* (8.9%) and *K. pneumoniae* (7.4%), respectively. Moreover, *S. aureus*, *P. aeruginosa*, *P. mirabilis* and *K. pneumoniae* were the microorganisms most frequently found with other bacteria in polymicrobial associations. The most common polymicrobial association was between *S. aureus* and *P. aeruginosa*, which was observed in 10 cases.

### 2.1. Microbial Drug-Resistance Profiles

Of the total of 135 isolates, 92 (68.2%) were antibiotic-sensitive isolates (Not-MDRO) and 43 (31.8%) were classified as MDROs. The antibiotic susceptibility patterns of not-MDRO isolates are summarized in Table 1 and Table 2, respectively. Methicillin-sensitive *S. aureus* (MSSA) were 70% to 100% responsive to all tested antibiotics, showing a 69.6% resistance only to benzylpenicillin. *P. aeruginosa* was totally susceptible to amikacin, ceftazidime, imipenem and meropenem, while, in 14.3% of cases, it showed resistance to ciprofloxacin and gentamicin. *K. pneumoniae* had a 50% resistance to gentamicin, the 3rd and 4th generation cephalosporins, amoxicillin/clavulanic acid, carbapenems and piperacillin/tazobactam, while amikacin was the only effective antimicrobial agent against this species. Carbapenem, aminoglycoside, the 3rd and 4th generation cephalosporins and nitrofurantoin had the same activity against *E. coli*; in fact, 100% of the bacterial species was susceptible to these agents. *P. mirabilis* showed high resistance to the antimicrobial agents tested; it was 100% sensitive only to ertapenem and meropenem. Table 1 and Table 2 summarize the profile of antibiotic resistance of all the other Gram-negative bacilli and Gram-positive cocci, respectively.

**Figure 2:**
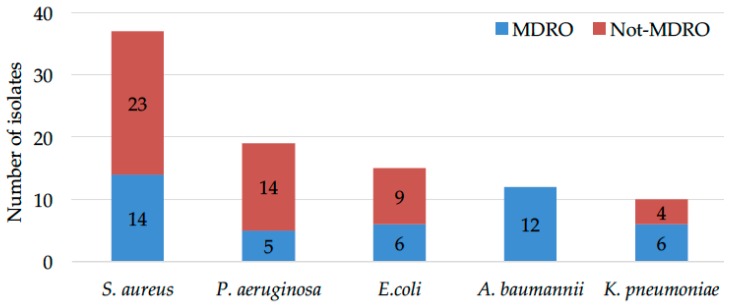
Quantity of not-MDRO (red) compared to its respective counterparts MDRO (blue): MRSA versus MSSA, MDRPA versus *P. aeruginosa*, ESBL-producing *E. coli* and *K. pneumoniae* versus *E. coli* and *K. pneumoniae*, respectively, and *A. baumannii*.

The assessment of MDROs (Figure 2) revealed that, within the 37 isolates of *S. aureus*, 14 (37.8%) were MRSA and 23 methicillin-sensitive *S. aureus* (MSSA). In particular, MRSA could be identified in 10.4% of the totality of the microorganisms.

Of the 19 *P. aeruginosa* isolates, five were MDRPA representing 26.3% of the *P. aeruginosa* isolates and 3.7% of the microorganisms analyzed. Among the 15 isolates of *E. coli*, six (40%) were ESBL-producers accounting for 4.4% of all microorganisms isolated in the ulcerations. *A. baumannii* was isolated from 12 samples (8.9%) and, in all cases, was classified as MDRO. Regarding the 10 *K. pneumoniae* isolates, six (60%) were ESBL-producers, resulting in 4.4% of all the identified microorganisms. As expected, MRSA were 100% resistant to benzylpenicillin and oxacillin. Conversely, they were sensitive (100%) to tigecycline, linezolid and vancomycin, while more than 40% were resistant to clindamycin, 50% resistant to erythromycin and gentamicin and 64.3% resistant to levofloxacin, respectively. Of note, we have observed the presence of three *S. aureus* strains (1 MSSA and 2 MRSA) identified as vancomycin resistant. In those cases, the vancomycin susceptibility profile was further verified by the minimal inhibitory concentration methods as suggested by the EUCAST clinical breakpoint tables. This latter testing revealed that all the *S. aureus* strains, previously identified as vancomycin resistant by the automated antimicrobial susceptibility assessment, were, in fact, vancomycin susceptible, scoring a minimum inhibitory concentration (MIC) < 1 μg/mL. These results support previous reports pointing to the risk of significant discordances in vancomycin resistance profiling as assessed by different susceptibility tests [34].

Table 3 illustrates the drug resistance profiles of all MDROs. MDRPA showed between 80% and 100% of resistance to amikacin, cefepime, ciprofloxacin, gentamicin and tobramycin. Moreover, it was resistant (from 20% to 40%) to carbapenems (meropenem and imipenem) and, in 60% of the cases, to ceftazidime, being sensitive only to colistin. *A. baumannii* showed 100% resistance to amoxicillin-clavulanic acid, the 3rd and 4th generation cephalosporins and fosfomycin. More than 70% resistance was noted to gentamicin, imipenem, trimethoprim-sulfamethoxazole and ciprofloxacin being susceptible only to colistin.

Both ESBL-producing *E. coli* and *K. pneumoniae* showed 100% resistance to the 3rd and 4th generation cephalosporins. Moreover, ESBL-producing *E. coli* was resistant (100%) to gentamicin and ciprofloxacin and sensitive (100%) to carbapenems, colistin, fosfomycin, nitrofurantoin and tigecycline, respectively. ESBL-producing *K. pneumoniae* showed a high resistance (83.3%) to amoxicillin-clavulanic acid, ciprofloxacin, piperacillin-tazobactam and trimethoprim-sulfamethoxazole, and, in addition, it was also resistant (50%) to colistin.

### 2.2. Assessment of Microbial Biofilm Production

**Figure 3:**
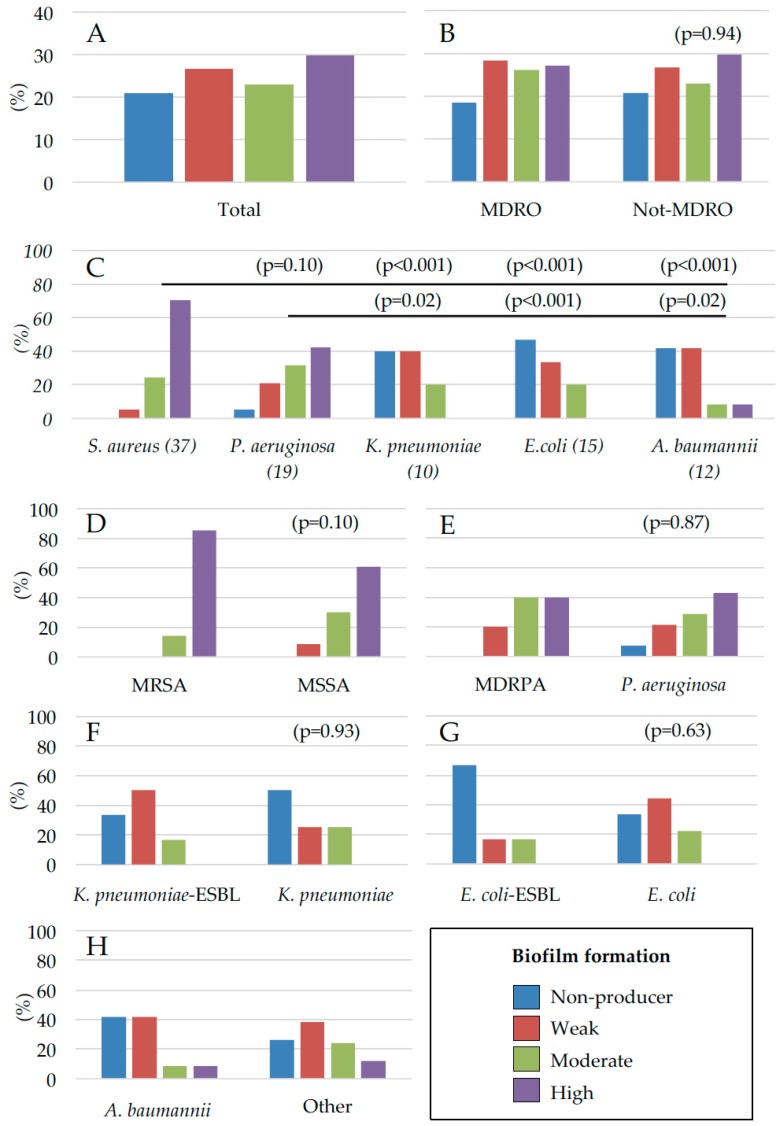
Biofilm production of bacterial isolates from patients with chronic skin ulcers. (**A**) biofilm production of the total bacterial isolates; (**B**) of MDRO and not-MDRO; (**C**) of the different bacterial species and according to multidrug resistance profile: (**D**) MRSA (*n* = 14) vs. MSSA (*n* = 23); (**E**) MDRPA (*n* = 5) vs. *P. aeruginosa* (*n* = 14); (**F**) *K. pneumoniae* ESBL (*n* = 6) vs. *K. pneumoniae* (*n* = 4), *E. coli* ESBL (*n* = 6) vs. *E. coli* (*n* = 9), and *A. baumannii* (*n* = 12) compared with the rest of isolates (*n* = 42). Biofilm formation was assessed by the cBRT and clinical isolates were classified as non-producers, weak, moderate and high biofilm producers. All results expressed as percentage of strains with the specific biofilm-forming ability.

The ability to produce biofilm was evaluated in all 135 bacterial isolates, including both MDRO and not-MDRO species, using the clinical BioFilm Ring Test (cBRT) [35]. Results showed that 79.3% of the isolates were able to produce biofilm (Figure 3A). Of the 43 MDROs analyzed, the biofilm-producing strains accounted for 74%, whereas, among the 92 not-MDROs analyzed, 86% were capable of forming biofilm (Figure 3B). Comparable profiles of biofilm production were observed in both MDRO and not-MDRO species (Figure 3B) and the statistical analysis revealed no significant differences between groups (*p* = 0.94).

Moreover, the biofilm profiling at the level of single bacterial species revealed that 94.6% of *S. aureus* isolates and 73.7% of the *P. aeruginosa* strains were moderate/high biofilm producers, respectively (Figure 3C). Conversely, only 60.0% of *K. pneumoniae* ESBL strains were found to produce biofilm and none of them was a high biofilm producer. Among the 12 *A. baumannii* strains, 58.3% were found to produce biofilm. Finally, *E. coli* was found to be the weakest biofilm producer with only 53.3% of the strains capable of forming biofilm (Figure 3C). Globally, *S. aureus* and *P. aeruginosa* showed a comparable ability to form biofilm (*p* = 0.1) and this ability was significantly higher with respect to *K. pneumoniae*, *E. coli* and *A. baumannii* (*p* < 0.05), respectively (Figure 3C).

Comparative analysis performed to evaluate different abilities to produce biofilm of the MRSA and not-MRSA, at the level of single bacterial species (Figure 3D–H), showed no significant differences between groups (*p* > 0.05). Of note, the vast majority of the bacterial isolates, present as single colonizers, were moderate/high biofilm producers. In particular, from the 45 ulcerative lesions colonized by single bacterial species, weak biofilm producers were found only in six cases (13.3%), while in the remaining 41 cases (86.6%), colonization was sustained by moderate/high biofilm-producers. Notably, all the strains, either belonging to MDRO or not-MDRO groups, identified as biofilm non-producers, were always associated in polymicrobial colonization with moderate/high biofilm producer strains.

## 3. Discussion

Data from this study show that Gram-negative bacteria were the most common colonizing species in chronic ulcerations, accounting for up to 60.8% of the isolates, and that MDROs accounted for the 31.8% of the total microbial isolates. Nevertheless, considering the individual bacterial species, *S. aureus* was the most common pathogen, found in 27.4% of the lesions. These data are consistent with previous studies describing a rate of colonization between 10% and 40%, with approximately 20% of the human population permanently colonized in their noses and another 60% of individuals considered intermittent carriers [36,37,38,39,40,41]. Higher carrier rates, however, were described in hospitalized patients or in patients with atopic dermatitis [40,41]. Among the 37 isolates of *S. aureus*, 14 were MRSA (37%), representing 10.3% of all microorganisms isolated. This suggests a higher frequency of MRSA, with respect to all *S. aureus* isolation in our hospital, as compared with previous studies [37]. In fact, to limit the spread of *S. aureus*, it has been recently activated in our institution a novel screening protocol for nasal MRSA detection, since *S. aureus* nasal carriage is a recognized risk for either nosocomial or community-acquired infections as well as presenting a high risk for ulcer colonization [42,43,44].

Apart from benzylpenicillin, clindamycin, erythromycin, MSSA showed a sensitive profile with 80% to 100% of the antibiotics tested. Conversely, MRSA showed high levels of antimicrobial resistance and the most effective agents were found to be linezolid and tigecyclin.

In addition, we found an apparent gender-specific preference in ulcers colonization by *S. aureus*. In fact, of the 37 *S. aureus* isolated, 24 originated from male patients and only 13 from female patients. This result is in contrast with other studies that reported a preferential colonization of *S. aureus* in females, and further suggests reconsidering possible differential gender-specific habits such as body hygiene [45]. Although *S. aureus* was the most prevalent individual microbial species, Gram-negative bacteria were the most abundant (60.8%) colonizing pathogens. Among them, *P. aeruginosa* was the second-most prevalent species, accounting for up to 14.1% of all microbial isolates. Many studies showed a large variation in the incidence of *P. aeruginosa* in ulcers, spanning from 14.6% to 34.1% [37,38,46]. One explanation for this high variability can be ascribed to the polymicrobial nature of wound communities and to the fact that *P. aeruginosa* preferentially localizes deeper in the wound tissue, thus resulting in being more difficult to isolate [47,48]. Despite *P. aeruginosa* is considered an easy-to-culture bacterium, in several cases of culture failure, molecular based techniques were effective at detecting this microorganism [49]. Thus, the isolation of *P. aeruginosa* by traditional culturing might be somehow limited, further raising the issue on the effectiveness of swabs for the detection of this bacterium. In this study, *P. aeruginosa* was detected in 14 cases of multiple colonizations, and, in 10 of these events, it was found to be associated with *S. aureus*.

Differently from *P. aeruginosa* isolates, MDRPA showed a much higher resistance to aminoglycosides (amikacin, gentamicin, tobramycin), cephalosporins and ciprofloxacin, and displayed a 20% to 40% resistance to carbapenems, being 100% sensitive only to colistin.

The other Gram-negative bacteria isolated include *P. mirabilis* (11.9%), *E. coli* (11.1%), *A. baumannii* (8.9%) and *K. pneumoniae* (7.4%). We noted that a considerable percentage of these species showed high resistance to the antibiotics tested. In particular, 100%, 60%, 40% and 26.3% of *A. baumannii*, *K. pneumoniae*, *E. coli* and *P. aeruginosa*, respectively, had an MDRO phenotype. ESBL-producing *E. coli* showed a 100% resistance to the 3rd generation (cefotaxime, ceftazidime) and the 4th generation (cefepime) cephalosporins, to ciprofloxacin and to gentamicin, but it was highly susceptible (70–100%) to all other antibiotics tested (with the exception of trimethoprim/sulfamethoxazole). ESBL-producing *K. pneumoniae* was resistant (100%) to the 3rd and 4th generation cephalosporins, and showed also 83.3% resistance to amoxicillin/clavulanic acid, ciprofloxacin, piperacillin/tazobactam and trimethoprim/sulfamethoxazole. Carbapenem and amikacin were the only effective antimicrobial agents against this organism. Colistin represented the only effective drug against *A. baumannii*, which is the species that showed the highest resistance to the panel of antibiotics tested, as compared with all other MDROs.

Although usually classified as non-MDRO, *P. mirabilis* and *M. morganii* showed a high level of beta-lactams resistance, an aspect which is rarely taken into consideration [50]. *P. mirabilis* is generally susceptible to β-lactams, due to the lack of a chromosomally encoded AmpC cephalosporinase. However, sporadic acquisition of different types of β-lactamases through plasmid-mediated mechanisms has been reported [51,52,53]. The individuals included in this study were all chronically colonized by different bacteria (including MDRO and non-MDRO), growing within a biofilm matrix and exposed to repeated antimicrobial treatments. Thus, it is tempting to speculate that such biofilm-endowed, polymicrobial colonization may have provided a most favorable environment for the exchange of plasmid-mediated antimicrobial resistance. On the other hand, *M. morganii* is also not generally classified as ESBL. Nevertheless, it has a known capability to express an inducible, chromosomally encoded, AmpC beta-lactamase with a high catalytic activity on classical penicillins, cefoxitin, narrow-spectrum cephalosporins, and cefotaxime, a property, which is consistent with the susceptibility profile presented in Table 1 [54].

It has been reported that biofilm production, particularly by MDROs, may play a relevant role in the pathogenesis of chronic wounds, considering its effects on the antibiotic resistance and the ensuing limitation of therapeutic options [16,22]. In the present study, we have found a strong propensity at biofilm formation by most microbial isolates. In fact, we found that up to 80.5% of the microbial strains analyzed were able to produce biofilm. These data are in accordance with previous reports, which described percentages of biofilm-producing bacteria between 60% and 90% in chronic wounds [22,47,49,55,56]. In particular, of the 135 strains analyzed 27.5% were classified as high, 26% as moderate, and 29.2% as weak biofilm producers, respectively. However, we found a comparable ability to produce biofilm in both MDRO (74.4%) and Not-MDRO (78.9%) strains, with no significant differences between the two groups (*p* = 0.94).

The role of biofilm in facilitating the emergence of resistance to antibacterial agents is still debated. However, it is largely recognized that a mature biofilm matrix may allow bacteria to elude the host immune response while forming a barrier against most conventional antimicrobial treatments. In addition, other biofilm-related mechanisms of drug resistance should be considered. For instance, the horizontal gene transfer is increased in biofilm growing bacteria in vitro, and this process is considered to be responsible for the acquisition of antibiotic resistance in vivo, both at single species and multispecies levels [57,58,59]. Enhanced rates of conjugation have been also reported in the biofilm of enterococci and Pseudomonas spp. [60,61]. Increased mutation frequencies have been described in biofilm cultures of *P. aeruginosa*, *S. aureus*, *Staphylococcus epidermidis* and *Streptococcus pneumoniae*, suggesting that the biofilm matrix provide a favorable environment to promote mutational resistance to antibiotics [62,63,64]. Moreover, bacterial biofilm appears to promote the maintenance of plasmids in the regions dominated by persister cells, that is, the residual bacteria present in the biofilm matrix as a dormant or non-growing microbial fraction, which is excluded by processes of competition and therefore able to preserve episomal factors [65]. On the other hand, it has been suggested that the slow diffusion of antibiotic drugs within the biofilm matrix might sustain the development of multidrug resistance, possibly by selecting highly tolerant strains transiently exposed to sub-inhibitory concentrations of antimicrobial drugs [59,66,67]. Nevertheless, some experimental evidence indicates that fluoroquinolones, tetracycline, rifampin, daptomycin and vancomycin can rapidly diffuse into the deeper levels of biofilm [68,69,70,71,72,73,74].

In this study, all of the 37 *S. aureus* isolates, including both MRSA and MSSA, showed a high level of biofilm production with no significant differences in the level of biofilm formation between groups (*p* = 0.10). This result is in accordance with previous reports, which failed to find any association between methicillin resistance and an increased ability to produce biofilms [26,29,75,76,77,78]. Nevertheless, other authors described a significantly higher rate of biofilm formation in *S. aureus* strains with greater multidrug resistance as compared with more susceptible strains [27]. Specifically, MRSA isolates were found to display higher adhesion properties than MSSA strains on bronchial epithelial cells and the expression of functional penicillin binding protein 2a (PBP2a), which is responsible for methicillin resistance, correlated with increased catheter adhesion ability [79,80]. In *S. aureus*, the acquisition of methicillin resistance has pleiotropic effects by reducing toxin production, altering biofilm formation and eventually reducing virulence [79]. The large number of results, often conflicting, on the adherence ability of the MRSA and MSSA strains may be associated with different strategies adopted by *S. aureus* in response to the environment, according to various sites of isolation. In health care-associated infections, MRSA has been found to exert an attenuated virulence with respect to community-associated infections by MRSA (CA-MRSA) strains [81]. This difference may reflect an adaptation to healthcare settings and to more vulnerable patients, whereas the equilibrium between antibiotic resistance and virulence observed in CA-MRSA appears more consistent with the need to infect healthy individuals [79,81]. Our results are representative of a specific court of patients where chronic ulcers more likely offer a selective advantage and an ideal environment for biofilm-producing *S. aureus*, independently from the acquisition of multidrug resistance.

Biofilm is considered an important virulence factor in several cases of infection/colonization by *P. aeruginosa* [82]. This is also confirmed by our study revealing that 100% of the MDRPA strains and 92.9% of the more susceptible *P. aeruginosa* isolates were able to produce biofilm. Comparison of biofilm production amongst *P. aeruginosa* clinical isolates showed no association between the multidrug resistance phenotype and the ability to produce biofilm (*p* = 0.87). Nevertheless, in previous reports, dealing with a larger number of *P. aeruginosa*, propensity to form biofilm was found significantly higher in MDRPA as compared to susceptible isolates deriving from infected wounds and from contaminated contact lens [29,83,84]. Here, we found that *S. aureus* (*p* < 0.01) and *P. aeruginosa* (*p* = 0.02) were frequently found in association, and this result is consistent with previous studies reporting that the dual infection causes a more severe patient outcome than an infection sustained by a single microorganism [5,37,42,47,85,86,87]. Generally, the dual infection *S. aureus*/*P. aeruginosa* causes the worsening of the outcome in patients with chronic wounds than single infections [85,86,88]. The interactions between *P. aeruginosa* and *S. aureus*, studied in an “in wound-like” model, demonstrated that *P. aeruginosa* and *S. aureus* tend to coexist stably and that this association provides a mutual benefit in terms of increased antibiotic tolerance [87].

Second only to *P. aeruginosa* among the nosocomial, aerobic, nonfermentative, Gram-negative bacilli, *A. baumannii* can harbour and transfer diverse antibiotic resistance determinants [89]. *A. baumannii* is recognized as an important multidrug resistant pathogen causing a major threat for hospitalized patients and biofilm is repeatedly implicated as an important determinant of virulence [28]. In our study, 58.3% of the *A. baumannii* were able to produce biofilm, a significantly low percentage compared with those observed for *S. aureus* and *P. aeruginosa* but in accordance with previous studies showing that 50–63% of the *A. baumannii* isolates were able to form biofilm [28,90,91]. Interestingly, five out of 12 strains of *A. baumannii* were classified as non-biofilm producers and those strains were always associated in multiple colonizations with biofilm-producing isolates. In addition, only a small number of *A. baumannii* isolates (16.3%) were classified as moderate/strong biofilm producers. These results suggest that, for *A. baumannii*, antibiotic resistance is key for the successful colonization of patients with chronic ulcers and that biofilm-producing polymicrobial colonization may increase the environmental resilience. Thus, it remains an open question whether *A. baumannii* biofilm production and the acquisition of multiple resistances coexist under the same selective pressure driven by the hospital environment. It has been reported that the acquisition of the MDR phenotype in *A. baumannii* occurs at the expense of biofilm formation, whereas, in the non-MDR isolates, biofilm production has a more important role in the bacterial persistence [92]. On the other hand, several authors dispute the existence of a possible relationship between biofilm formation and MDR phenotype in *A. baumannii*, based on the notion that weak biofilm producers often display a high level of multidrug resistance [92,93]. Consistent with these observations, high biofilm-producing *A. baumannii* was found to be sensitive to several antibiotics, suggesting that biofilm may not be necessary for a successful dissemination of *A. baumannii* within the hospital setting [94].

Likewise, considering *K. pneumoniae*, the percentage of biofilm producers was significantly lower than that of *S. aureus* and *P. aeruginosa* (*p* < 0.05). Specifically, 66.7% and 50% of *K. pneumoniae*-ESBL and *K. pneumoniae*, respectively, were able to form biofilm with no significant differences in the overall level of biofilm production (*p* = 0.93). Additionally, none of the *K. pneumoniae*-ESBL and *K. pneumoniae* isolates were found to be strong biofilm producers and all the isolates were detected in polymicrobial colonizations. Previous in vitro studies revealed that approximately 40–50% of *K. pneumoniae* clinical isolates, derived from different materials, were able to form biofilm [34,95]. The existence of a significant correlation between antibiotic resistance and the level of biofilm production has been reported in *K. pneumoniae* isolates deriving from sputum and urine, but limited data are available from strains isolated from wounds [96]. *E. coli*, irrespective from the multidrug resistance phenotype, was classified as the weakest biofilm producer. The percentage of strains able to form biofilm was 33.3% and 66.7% for *E. coli*-ESBL and *E. coli*, respectively. Despite the level of biofilm producers being higher within the *E. coli* isolates as compared with their multidrug resistant counterpart, the difference was not statistically significant (*p* = 0.63), possibly due to the limited number of strains.

It is important to highlight that those bacterial strains classified as non-biofilm producers were always found to be in association in polymicrobial colonization with biofilm producer strains. Isolation of multiple species of bacteria from a wound specimen is common in many studies [97]. This notion is further supported by previous microbiome studies of the wound that revealed the presence of complex interspecies interactions that can affect the healing process [98]. Pyrosequencing techniques showed a large microbial diversity within wounds with more than 100 different bacterial genera belonging to strict and facultative anaerobes generally unidentified by standard culturing techniques [21,99]. Our observations provide novel information about the complexity of the ulcer microenvironment, which appears to be invariably characterized by a strong biofilm production sustained by either non-MDRO or MDRO strains, often supported by polymicrobial associations. This notion reinforces the concept that bacterial species may act synergistically during the colonization of ulcers and further suggests that the formation of a structured biofilm matrix is key in the “critical colonization” process and in the promotion of chronicity. Indeed, biofilm production may provide key virulence properties to colonizing bacteria including protection from the host immune response as well as by making the microorganism more generically tolerant to a broad range of antibiotics, as compared to non-biofilm producers. Furthermore, such biofilm-endowed mixed colonization might also promote the acquisition of novel drug resistance properties favoring the exchange of diffusible genetic materials.

Knowledge of the characteristics and properties of such polymicrobial colonization provides novel interpretation to the “critical colonization” concept, which may help in supporting a more thoughtful approach at the clinical management of chronic ulcers and future therapeutic strategies aimed at attacking the biofilm matrix.

## 4. Materials and Methods

The Central Ethics Committee I.R.C.C.S. Lazio, section of the Istituti Fisioterapici Ospitalieri in Rome, approved this study (Prot. CE/1016/15—15 December 2015, trials registry N. 730/15).

### 4.1. Inclusion Criteria

The study included only those patients with colonized skin ulcers according to specified guidelines [12,100,101,102,103,104]. Briefly, an ulcer was classified as chronic when it persisted since at least 3 months [104]. Exclusion criteria were the presence of fungi in a polymicrobial infection and a pre-existing systemic antimicrobial treatment and/or local antiseptic therapy within 2 weeks prior to ulcer sampling.

### 4.2. Sample Collection

Sample collection was performed from a total of 87 patients with colonized skin ulcers attending the Istituti Fisioterapici Ospitalieri (IFO), which include the San Gallicano Dermatologic Institute and the “Regina Elena” National Cancer Institute, in Rome, Italy, from January 2013 to December 2015. Sample collection was performed by commercially available sterile swabs (COPAN swabs, Brescia, Italy), according to existing departmental guidelines. Only one swab per patient was collected after careful wound cleaning and debridement in order to prevent surface contamination.

The samples were processed within two hours after collection for culture analysis and biofilm assessment.

### 4.3. Bacterial Identification

Swabs were immediately inoculated on Columbia CNA Agar with 5% Sheep Blood (bioMérieux, Bagno a Ripoli, Italia), MacConkey Agar (bioMérieux, Bagno a Ripoli, Italia), Sabouraud Agar with gentamicin (bioMérieux, Bagno a Ripoli, Italia) and *S. aureus* ID Agar (SAID; bioMérieux, Bagno a Ripoli, Italia) for the isolation and cultivation of Gram-positive and Gram-negative bacteria. Inoculated plates were incubated at 37 °C aerobically for 24 to 48 h. The chromogenic media SAID Agar was used for the initial identification of *S. aureus*. Additional confirmation tests for MRSA, such as agglutination tests for Penicillin-Binding Protein (PBP2, Oxoid, Basingstoke, UK) and cefoxitin screening, were performed. The automatic VITEK^®^ 2 (bioMérieux, Bagno a Ripoli, Italia) was used for the identification of the bacterial species and for the definition of the specific antimicrobial susceptibility tests [105], according to The European Committee on Antimicrobial Susceptibility Testing (EUCAST Clinical Breakpoint Table v 7.1). In case of vancomycin resistant *S. aureus* strains, identified by VITEK^®^ 2 (bioMérieux, Bagno a Ripoli, Italia), the antimicrobial susceptibility was further verified by the minimal inhibitory concentration methods (Thermo Scientific, Basingstoke, UK).

MDROs were defined as MRSA, ESBL-producing *K. pneumoniae* and *E. coli*, *P. aeruginosa* MDR and *A. baumannii* MDR, according to the Healthcare Infection Control Practices Advisory Committee (HICPAC) guidelines [106] and the local infection control committee. Phenotypic detection of ESBL producers was further defined using disk approximation tests (Thermo Scientific, Basingstoke, UK) as previously described [107,108]. *P. aeruginosa* was defined MDR when resistant to at least one antimicrobial agent of at least three categories of antimicrobial agents selected from the following: aminoglycosides (gentamicin, tobramycin, amikacin, netilmicin), antipseudomonal carbapenem (imipenem, meropenem, doripenem), antipseudomonal cephalosporin (ceftazidime, cefepime), antipseudomonal fluoroquinolones (ciprofloxacin, levofloxacin), antipseudomonal penicillins + β-lactamase inhibitors (piperacillin-tazobactam, ticarcillin-clavulanic acid), monobactams (aztreonam), phosphonic acids (fosfomycin) and polymyxins (colistin, polymyxin B). *A. baumannii* was defined as MDR when resistant to at least one antimicrobial agent of at least three categories of antimicrobial agents selected from the following: aminoglycosides (gentamicin, amikacin, netilmicin), carbapenem (imipenem, meropenem), cephalosporin (cefotaxime, ceftazidime, cefepime), fluoroquinolones (ciprofloxacin, levofloxacin), and penicillins + β-lactamase inhibitors (ampicillin-sulbactam, piperacillin-tazobactam, ticarcillin-clavulanic acid). The MDRO were frozen and stored at −80 °C.

### 4.4. Evaluation of Biofilm Production

Biofilm production was evaluated by the clinical BioFilm Ring Test (cBRT) [35]. Briefly, overnight culture of bacteria grown on specific agar plate was used to inoculate, 2 mL of 0.45% saline solution (AirLife, Carefusion, San Diego, CA, USA) to the equivalent of 1.0 ± 0.3 McFarland turbidity standard. The bacterial suspension was used to inoculate a 96-well polystyrene plate with 200 μL/well. The test was performed using toner solution (TON004) (Biofilm Control, Saint Beauzire, France) containing magnetic beads 1% (*v*/*v*) mixed in Brain Heart Infusion medium (BHI, Difco, Detroit, MI, USA). Sample dilutions (10-fold serial dilutions), were done in a volume of 200 μL BHI/TON mix.

One or more laboratory strains were included in each plate as standard reference and quality control. A well containing the BHI/TON mix without microbial cells was used as negative control in each experiment.

After five hours of incubation at 37 °C without shaking (static culture), wells were covered with a few drops of contrast liquid (inert opaque oil used) placed for 1 min on the block carrying 96 mini-magnets (Block test) and scanned with a specifically designed plate reader (Pack BIOFILM, Biofilm Control, Saint Beauzire, France). The adhesion strength of each strain was expressed as BioFilm Index (BFI) Each microbial culture was analyzed in duplicate and experiments were repeated at 3 times for each strain.

### 4.5. Statistic Analysis

Statistical analysis was performed using the chi-square test for linear trend and adjusted for multiple comparisons when appropriate. Data analysis was calculated using one-way ANOVA. *p*-values of 0.05 or less were considered statistical significant. All statistical analyses were performed using IBM SPSS v.21 statistics software (IBM, Chicago, IL, USA).

## 5. Conclusions

Epidemiology of MDROs is extremely complex and multifactorial, varying temporally and geographically, and further depending on patients’ characteristics. In this study on patients with chronic skin ulcers, MDRO frequencies accounted for the 31.8% of the total isolates. Our results showed a high prevalence of Gram-negative bacteria (60.8%) and, among this group, *P. aeruginosa* was the most frequent pathogen isolated. Nevertheless, considering the individual bacterial species, *S. aureus* was found to be the most common pathogen with linezolid and tigecycline being the most effective antimicrobials. Globally, carbapenem and colistin were the most effective antimicrobial agents against all Gram-negative MDRO.

Up to 80.5% of the microbial strains analyzed were able to produce biofilm. Notably, non-biofilm producers or poor producers were always found to be in association with biofilm producers, suggesting that bacterial species growing in polymicrobial colonization may act synergistically, forming biofilm-embedded communities mutually interacting in the promotion of wound chronicity. These results further point to biofilm formation as a key determinant of pathogenicity, capable of promoting a successful, persistent colonization of the wound site, independently from the acquisition of multidrug resistance.

## Figures and Tables

**Table 1:** Antibiotic resistance pattern of Gram-negative bacilli not MDR (% of resistance). *n* represents the number of samples. Blank: data not available.

Drug	*E. coli* *n* = 9	*K. pneumoniae* *n* = 4	*P. mirabilis* *n* = 16	*E. cloacae* *n* = 5	*C. freundii* *n* = 2	*M. morganii* *n* = 2	*P. aeruginosa* *n* = 14
Amikacin	0	0	43.8	0	0	0	0
Gentamicin	0	0	75	0	0	50	14.3
Tobramycin						50	11.1
Ertapenem	0		0		0	0	
Imipenem	0	0		0	0		0
Meropenem	0	0	0		0	0	0
Cefepime	0	0	25	0	0	0	7.1
Cefotaxime	0	0	62.5	0	0	100	
Ceftazidime	0	0	62.5	0	0	100	0
Nitrofurantoin	0		100				
Amoxicillin/clavulanic acid	22.2	0		100	100		100
Piperacillin/Tazobactam		0		0	0		
Colistin	0	0	100		0	100	7.1
Ciprofloxacin	22.2	0	68.8	0	0	66.6	14.3
Levofloxacin						50	
Trimethoprim/sulfamethoxazole	55.5	0	75	20	0	50	100
Tigecycline			100			100	

**Table 2:** Antibiotic resistance pattern of Gram-positive cocci not MDR (% of resistance). MSSA = methicillin-sensitive *S. aureus*. *n* represents the number of samples. Blank: data not available.

Drug	MSSA *n* = 23	*E. faecalis* *n* = 6	*S. agalactiae* *n* = 2
Gentamicin	18.2		
Gentamicin High Level Resistance		40	
Streptomycin High Level Resistance		80	
Imipenem		0	
Teicoplanin	4.3	0	0
Vancomycin	0	0	0
Clindamycin	26.1	100	100
Daptomycin	0		
Erythromycin	31.8	66.7	
Nitrofurantoin		0	0
Linezolid	0	0	0
Ampicillin/sulbactam		0	
Benzylpenicillin	69.6		0
Oxacillin	0		
Levofloxacin	8.7	50	0
Moxifloxacin			0
Trimethoprim/sulfamethoxazole	0	100	0
Tetracyclin	8.7		100
Fusidic Acid	0		
Tigecycline	0	0	0

**Table 3:** Antibiotic resistance pattern of MDROs (% of resistance). Blank: data not available. MRSA = methicillin-resistant; ESBL = positive to the production of extended-spectrum beta-lactamase; MDRPA = multidrug resistant *P. aeruginosa*. *n* represents the number of samples.

Drug	MRSA *n* = 14	*A. baumannii* *n* = 12	*E. coli* ESBL *n* = 6	*K. pneumoniae* ESBL *n* = 6	*MDRPA* *n* = 5
Amikacin			16.7	33.3	80
Gentamicin	50	75	100	66.7	100
Tobramycin					100
Ertapenem			0		
Imipenem		75	0	33.3	20
Meropenem			0	33.3	40
Cefepime		100	100	100	80
Cefotaxime		100	100	100	
Ceftazidime		100	100	100	60
Teicoplanin	7.1				
Vancomycin	0				
Clindamycin	42.9				
Daptomycin	7.7				
Erythromycin	50				
Nitrofurantoin			0		
Linezolid	0				
Amoxicillin/clavulanic acid		100	33.3	83.3	100
Benzylpenicillin	100				
Oxacillin	100				
Piperacillin/Tazobactam			20	83.3	
Colistin		0	0	50	0
Ciprofloxacin		91	100	83.3	80
Levofloxacin	64.3				
Moxifloxacin	15.4				
Trimethoprim/sulfamethoxazole	14.3	75	66.7	83.3	100
Tetracyclin	28.8				
Fosfomycin		100	0	66.7	
Fusidic Acid	7.1				
